# Effect of a Lactobacilli-Based Direct-Fed Microbial Product on Gut Microbiota and Gastrointestinal Morphological Changes

**DOI:** 10.3390/ani14050693

**Published:** 2024-02-23

**Authors:** John I. Alawneh, Hena Ramay, Timothy Olchowy, Rachel Allavena, Martin Soust, Rafat Al Jassim

**Affiliations:** 1 School of Veterinary Science, University of Queensland, Gatton, QLD 4343, Australia; r.allavena@uq.edu.au; 2 Faculty of Veterinary Medicine, University of Calgary, Calgary, AB T3R 1J3, Canada; henaramay@gmail.com (H.R.);; 3 Terragen Biotech Pty Ltd., Coolum Beach, QLD 4573, Australia; martins@terragen.com.au; 4 Queensland Alliance for Agriculture and Food Innovation, St Lucia, QLD 4072, Australia; r.aljassim@uq.edu.au

**Keywords:** direct-fed microbials, GIT morphology, calves, live weight, diversity, microbiota

## Abstract

**Simple Summary:**

This study aimed to characterise and compare the development of gut microbiota in dairy calves from birth to weaning, focusing on the impact of a direct-fed microbial (DFM) product containing three strains of lactic acid bacteria (LAB). Forty-four Holstein-Friesian calves were randomly assigned to Treatment (TRT) and Control (CON) groups. TRT calves received a daily dose of the DFM, while CON calves received a placebo and served as the control. Faecal samples and necropsies were collected for analysis. TRT calves exhibited higher live weights at weaning and comparable average daily live weight gain and feed intake to CON calves. TRT calves also demonstrated greater weights of specific gut segments (duodenum, abomasum, reticulum) and enhanced rumen and intestinal development. The microbial diversity was more pronounced in the TRT group, with differences in the relative abundances of eight genera. This study suggests that supplementing with the LAB-based DFM positively influenced calfs’ weight, gut development, and microbial diversity. Further research is recommended to explore potential associations between DFM products and gut mucosa-associated microbiota.

**Abstract:**

The calf’s gastrointestinal tract (GIT) microbiome undergoes rapid shifts during early post-natal life, which can directly affect calf performance. The objectives of this study were to characterise and compare differences in the establishment and succession of GIT microbiota, GIT morphological changes, and the growth of dairy calves from birth until weaned. Forty-four newborn Holstein-Friesian calves were randomly selected and assigned to Treatment (TRT) and Control (CON) groups. The TRT group calves received a once-daily dose of a direct-fed microbial (DFM) liquid product containing *Lacticaseibacillus paracasei*, *Lentilactobacillus buchneri*, and *Lacticaseibacillus casei*, all formerly known as *Lactobacillus*. Fresh faecal samples were manually taken from the rectum of all calves, and gross necropsy was performed on the forestomachs and gastrointestinal tracts. Bacterial DNA was extracted from frozen faecal samples for 16S rRNA gene amplicon sequencing. Calves in the TRT group had greater live weights (*p* = 0.02) at weaning compared with calves in the CON group (mean = 69.18 kg, SD = 13.37 kg). The average daily live weight gain (ADG) and total feed intake were similar between the two groups. Calves in the TRT group had greater duodenum, abomasum, and reticulum weights (*p* = 0.05). Rumen and intestinal development (*p* < 0.05) and faecal microbial diversity (*p* < 0.05) were more pronounced in the TRT group. The relative abundances of eight genera differed (*p* < 0.001) between the groups. Supplementing calves with the LAB-based DFM increased live weight at weaning and had a more pronounced effect on the development of rumen and the gastrointestinal tract and on microbiota diversity and evenness. Future work is needed to better understand the potential association of LAB-DFM products on gut mucosa-associated microbiota.

## 1. Introduction

The colonisation of the gut microbiota of calves and the operation of the microbiota has been recognised as significant factors influencing their growth and development [1]. During early post-natal life, the colonisation of the gut by various microbial species plays a pivotal role in nutrient metabolism, immune system maturation [2,3,4], and overall health [5,6]. The microbial composition and diversity in the calf’s gastrointestinal tract undergoes dynamic changes, with initial colonisation by facultative anaerobes (i.e., *Lactobacillus*, *E. Coli*, and other genera of the *Enterobacteriaceae* family) [6,7], followed by the establishment of anaerobic bacterial populations (e.g., *Clostridium* spp., *Bifidobacterium* spp.) [8,9]. These microbiota alterations have been associated with shifts in energy extraction from feed, which can impact growth efficiency [10]. Furthermore, a balanced gut microbiome contributes to immune competence and defence against pathogens, ultimately influencing calves’ health [5,6,11] and growth rates [10]. Further insights into the interplay between calves’ gut microbiota during critical growth stages have the potential for devising targeted interventions aimed at enhancing animal health and welfare, as well as production efficiency.

The weaning age of calves holds a significant influence over subsequent productivity [12,13]. The timing for weaning [6,14] has been shown to influence the gut microbiota’s structure and the maturation of the calf’s gastrointestinal system as calves transition from a liquid milk diet to solid feed [6,15,16]. During this critical period, it has been shown that modification of calf feeding management could have prolonged effects on the gastrointestinal microbiota [17,18].

This dominance of *Lactobacilli* in the GIT ecosystem is of particular significance, as research has highlighted their pivotal role in modulating host defences [19,20]. Solid feed introduction triggers ruminal fermentative processes and enriches the indigenous microbiota, with a significant shift during weaning due to the alteration of nutrition sources that impact ruminal and intestinal microbiomes [1,21,22,23]. Rumen microbial fermentation products are pivotal for the development of rumen wall papillae, facilitating microbial colonisation as animals age. Recent studies indicate that a shift from aerobic to anaerobic microbes occurs around six weeks of age [14,24]. Within this context, the concept of utilising direct-fed microbials (DFMs) gains significance [25]. An effective DFM should ideally be tailored to support the proliferation of the indigenous microbiota that naturally inhabits the calf’s GIT [1,26]. This microbial intervention can potentially influence the trajectory of GIT development and microbial colonisation, thus influencing the calf’s overall health and growth [27]. Considering this, we hypothesised that the dairy calf’s enteric microbiota structure and function would change in response to DFM administered as a supplement in their milk replacer diet over the entire pre-weaning period. We also investigated a secondary hypothesis: that changes in the GIT morphology and calves’ growth would also occur in response to the earlier maturation of the GIT and the improved nutrient intake. Thus, the objective of this study was to characterise and compare differences in the establishment and evolution of GIT microbiota, GIT morphological changes, and the growth of dairy calves from birth to weaning in response to feeding a LAB-based DFM product as part of their daily milk diet.

## 2. Materials and Methods

### 2.1. Study Animals

This longitudinal study was conducted between June and October 2018 at the University of Queensland Gatton Commercial Dairy, Queensland, Australia (27.5636° S, 152.2800° E). The experimental protocol was reviewed and approved by the University of Queensland’s Animal Ethics committee (animal ethics approval No. SVS/128/18). A detailed description of the study’s methodology is described elsewhere [28]. Briefly, forty-four healthy newborn (male = 16, female = 28) Holstein-Friesian (HF; n = 26) and HF cross breed (n = 18) calves were randomly selected and enrolled. Following separation from their dams, calves were randomly assigned to individual pens in a well-ventilated, draft-free calf housing facility. Each calf was fed 2 L of high-quality colostrum (specific gravity of >1.050 [29] and IgG concentrations of >50 g/L [30]) via oral intubation within 8 to 12 h of birth [31]. The calves were provided with water (ad libitum), a calf-starter pellet (Calf Starter Crumble, Norco^®^, Brisbane, QLD, Australia; 20% crude protein, 8% crude fibre, 0.5% salt, 12.5 MJ/kg dry matter, 12% moisture/88% dry matter) and short chopped pasture hay from birth. The study animals were offered milk replacer (Norcovite, Norco^®^, Brisbane, QLD, Australia) mixed with water at the rate of 125 g/L at the same rate as the rest of the calves in the commercial dairy herd. Composition of the powdered calf milk replacer was 28% protein, 22% fat, 5% moisture, 40% lactose, and 95% dry matter. Calves were fed 4 L/d (divided over two meals) of milk replacer until three days of age. Thereafter, calves were fed milk replacer at 15% of live weight (LW), divided into two daily portions. The milk replacer was fed at 37 °C. The study animals were offered the same grain pellets as the rest of the calves in the commercial dairy herd. There were no feed additives or antibiotics added to the feed. Refused chaff and pellets were weighed and discarded every three days for each calf, and water was changed daily. Milk replacer feeding rate remained unchanged until calves were consuming at least 700 g of pellets with evidence of hay consumption for three consecutive days. At this time (approximately Day 42), calves were weaned off milk by being offered 2 L of milk only in the morning for two days, then offering 1 L each morning for three days. Weaning was considered successful if calves maintained feed intake and LW at the next weighing interval (by approximately Day 56). Calf live weights were recorded on Days 0, 14, 28, 42, and 56, and twice-daily calf health monitoring was performed by skilled workers (n = 6) throughout the study period. Using single-use disposable gloves, fresh faecal samples were collected directly from the rectum of all calves on Days 0 (before consumption of colostrum or milk replacer), 14, 28, 42, and 56, within 20 min of the morning feeding. Each sample was placed in a sterile plastic container and immediately placed and kept on ice until frozen at −80 °C.

### 2.2. Study Design and Animal Enrolment

This was a double-blinded, placebo-controlled study, with randomised design blocked on treatment groups (Control [CON] and Treatment [TRT] groups) to minimise the confounding effect of breed and age at enrolment. Sample size calculation was based on the following assumptions: an average live weight gain in the CON group of 0.8 kg/day with an average standard deviation of 0.2 kg/day. A total of forty-four calves were required to demonstrate a 25% difference (alpha = 0.05, power = 80%) in live weight change between the experimental groups. Prior to the start of the study, a list of all pregnant cows (n = 60) in the source herd was obtained. A simple randomisation without replacement technique was used to randomly assign treatment (CON or TRT) to the calves that were subsequently born to these cows. Treatments were administered by mixing 1 mL of the DFM supplement (liquid product containing *L. paracasei*, *L. buchneri*, and *L. casei* at a minimum of 10^9^ cfu of each) or placebo (as appropriate) with approximately 25% of a calf’s allotted milk replacer meal. After this treated milk was consumed, the remaining 75% of the meal was provided to the calf. All study personnel were blinded to the specific identity of the individual treatments (i.e., DFM vs. placebo) being administered to the study animals and to the treatment group assignments.

### 2.3. Bacterial DNA Extraction from Faecal Samples

Bacterial DNA was extracted from frozen faecal samples using the Quick-DNA™ Faecal/Soil Microbe Miniprep Kit (QIAGEN Chadstone, VI, Australia) following the manufacturer’s guidelines. DNA was evaluated based on optical densities at 230, 260, and 280 nm wavelength using a NanoDrop ND-1000 spectrophotometer (Thermo Scientific NanoDrop^TM^, Brisbane, QLD, Australia).

### 2.4. 16S rRNA Gene Amplicon Sequencing

16S rRNA gene amplicon sequencing of the gDNA extracted from each sample was conducted by the Australian Centre for Ecogenomics (ACE; St Lucia, QLD, Australia). Samples were submitted in 20 μL aliquots, with a minimum concentration of 5 ng/μL, accompanied by the quantification data. The V6–V8 variable region of the 16S rRNA gene was amplified using the following forward and reverse primers (with Illumina adaptors): 926F (5′-TCG TCG GCA GCG TCA GAT GTG TAT AAG AGA CAG AAA CTY AAA KGA ATT GRC GG-3′) and 1392wR (5′-GTC TCG TGG GCT CGG AGA TGT GTA TAA GAG ACA GAC GGG CGG TGW GTR C-3′) [32]. Paired-end sequencing (2 × 300 bp) was carried out by ACE using the Illumina MiSeq platform (Illumina, San Diego, CA, USA), and each sample was sequenced to a depth of 3 GB. All fastq files were trimmed to remove primer sequence using Cutadapt (Cutadapt, version 1.2.1). Filtered de-multiplexed reads were then analysed using the DADA2 pipeline plugin to resolve reads to high-resolution amplicon sequence variants (ASVs), which represent, as closely as possible, the original biological sequence of the sequenced amplicon [33]. Trimming was performed to remove poor-quality sequences using a sliding window of 4 bases, with an average base quality above 15 for sequence quality plots as guidance. All reads were then hard-trimmed to 220 bases, and any with less than 210 bases were excluded.

Multiple sequence alignment of ASV representative sequences was carried out using DADA2. FastTree V2.1 [34] software was then used to infer unrooted and subsequently rooted maximum likelihood phylogenetic trees representing the phylogenetic relatedness of ASVs. ASVs were taxonomically classified using a downloaded Naïve Bayes classifier, pre-trained on Silva database V13.1. Following taxonomic classification, ASVs comprising <3% of all reads, found in only one sample, or classified as mitochondria or chloroplast, were removed.

### 2.5. Gross Pathology and Histopathology Examination

After weaning (age 56 days), three Friesian male calves from the CON (n = 3) and TRT (n = 3) groups were randomly selected for postmortem. All postmortem examinations were carried out by the same pathologist from the Veterinary Laboratory Services, University of Queensland. At postmortem, the individual compartments of forestomachs (rumen, reticulum, and omasum) and the gastrointestinal tract (abomasum, duodenum, jejunum, ileum, caecum, and colon) were isolated and ligated. The oesophagus and rectum were tied proximally and distally, respectively, to avoid leakage of ingesta. The forestomachs and abomasum were isolated, and the visceral fat was removed with surgical scissors. The total weights of the forestomachs, abomasum, and intestine (duodenum, jejunum, ileum, caecum, and colon) were weighed when they were full of digesta and again when emptied. The rumen was dissected into its component parts, as was the gastrointestinal tract. Samples (2 cm × 2 cm, approximately) of the rumen (antrum, ventral, caudal, and dorsal sacs) were collected. Within 10 cm of the pylorus, 2 cm × 2 cm of duodenal (entire length) and jejunal segments (proximal, mid, and distal) were collected. Ileum was the last small intestine collected, and it was collected within 10 cm of the caecum. The entirety of the small intestine, caecum, and large intestine were weighed, and their individual lengths were measured with ingesta. Ingesta was removed and the structures were re-weighed. The entire rumen and intestinal tissues were fixed in ~4% formaldehyde solution for morphometric measurement. Concurrently, a gross necropsy examination was performed to evaluate sections of the gastrointestinal tract (forestomach, abomasum, duodenum, jejunum, ileum, caecum, and colon). If observed, a sample of any pathology or abnormality was collected. All tissue samples were immediately placed into cassettes, identified, and then placed in a ~10× volume of 10% neutral buffered formalin for a minimum of 48 h. The fixed samples were then trimmed, pressed, and processed in paraffin wax for serial recuts of 40-micron thickness. The slides were mounted and scanned to obtain an electronic image of the histological cuts. Samples of the rumen epithelium of the antrum, ventral sac, and ventral blind sac were used for determination of papilla mitotic rate, length, width, density, and surface area. In the intestine, villus length, width, and surface area were measured in the mucosa of the duodenum, proximal, mid- and distal jejunum, and ileum. Using Leica Image-Scope, histological samples were chosen based on systematic uniform random sampling (SURS) in a correct plane of a sample. The average length and width of the tissue were measured, and a sampling interval was chosen that captured approximately 20 measurements per slide per animal. With a pooled variance of 0.1, a power of 80%, and an alpha of 5%, the sample size for detecting a difference of 0.2 in villi measurements was 160 measurements from each group.

For the histological linear measurements of the sampled structures, the area of interest was assessed based on fractionator sampling, where rectangular sampling fields were chosen using systematic random sampling. For intestinal villi (caecum, jejunum, duodenum, ileum, colon) length and for abomasal thickness measurements, a section of 20–30 grids were chosen randomly over the area of the slide where the most measurable area was positioned. Every second square was selected, and those squares with full-thickness measurable villi were then selected for measurement. Any given villus was measured from the basement membrane to the villus tip, regardless of whether that basement membrane was outside the square (Figure S1). Forestomach (reticulum, rumen, omasum) linear measurements of the villi were obtained through the measurement of all intact villi to ensure the minimum sample number needed was attained (Figure S2). When required (n = 10), histological recuts were acquired to obtain an adequate number of measurements to meet sample size requirements.

Surface area measurements were acquired through the use of a Nikon™ Eclipse^®^ microscope linked to a Nikon™ viewing platform and Nis-Elements^®^ software to measure histology surface area. Using the 40× objective on both microscope and software, each villus or mucosal thickness was measured by adjusting the microscope slide on the microscope to the correct sample field. As the grid fractionator was not available as part of the Nikon software and microscope layout, the sample field was chosen at random, along with samples of intact tissue, for each sample on each slide. Each villus was meticulously outlined to ensure that the correct surface area measurement was obtained (Figure S2).

For larger villi or the fronds of the forestomach, multiple adjustments with the microscope were required to measure the entire surface area of the villi. For these larger measurements, the surface area was measured consecutively and added up to give the entire surface area of the frond/villi.

### 2.6. Alpha/Beta Diversity Analyses

Microbial diversity was assessed by calculating the following alpha diversity metrics: Shannon’s (Shannon’s DI) and Simpson’s (Simpson DI) Diversity Indices and observed ASVs. Evenness (a comparison of the relative abundance of each species in different samples) was calculated using Pielou’s Evenness (Pielou’s E). Compositional similarity/dissimilarity between samples (beta diversity) was estimated by generating weighted and unweighted UniFrac, Jaccard, and Bray–Curtis dissimilarity matrices for all pairwise sample comparisons. Compositional dissimilarity of samples was visualised using principal co-ordinates analysis (PCoA) of beta diversity distance matrices. Significant associations between alpha diversity metrics and metadata variables were tested using Kruskal–Wallis with Benjamin–Hochberg multiple test correction. Pairwise comparison of beta diversity distances between categorical metadata groups was analysed employing permutational multivariate analysis of variance (PERMANOVA), whilst significant correlations between numerical metadata categories and beta diversity distances were investigated using Mantel tests with 1000 permutations. To test for associations between longitudinal changes in alpha and beta diversity over time and for the different treatment groups we performed linear mixed-effects (LMEs) regression analysis. This accounted for subject-specific variation by using calf ID as a random effect, whilst allowing for identification of longitudinal differences in alpha/beta diversity due to treatment group by using that category as a fixed effect. The LME models were fitted with a first-order autoregressive correlation. Fitted residuals were assumed to follow a normal distribution with a mean of zero and a variance of σ^2^.

Differential ASV abundances at the genus level were compared among groups (CON vs. TRT) and calf age (Days 0, 14, 28, 42, and 56). This analysis was performed in DESeq2 using CON and TRT groups as a covariate and Benjamini–Hochberg (BH) adjustment for multiple tests [35]. Open-source software PICRUSt2 (phylogenetic investigation of communities by reconstruction of unobserved states) [35] and aldex2 package [36,37] in R [38] were used on the 16S rRNA gene sequencing data to predict functional genes of the classified members of the rumen and faecal microbiota resulting from reference-based OTU picking against the Greengenes database. Predicted genes were then hierarchically clustered and categorised under Kyoto Encyclopaedia of Genes and Genomes (KEGG) [39] orthologs (KOs) and pathways [40] (level 3).

### 2.7. Histopathology and Live Weight Data Analyses

First-order descriptive statistics were generated from continuous variables, while categorical variables were presented as counts and percentages. For histopathology data, Student’s *t*-test or Kruskal–Wallis test were used (as appropriate) to test if a given set of histopathology measurements were associated with treatment group. *p* values were adjusted for multiple comparison using Bonferroni correction method [41]. Applying Bonferroni correction, we strengthened the result reliability by rigorously adjusting *p*-values for multiple comparisons to minimise false positives [41]. The analysis was carried out using the epi.R package [42] in R [38].

A mixed-effects linear mode was fitted to the data to estimate calf’s live weight as a function of calf’s age (days), breed, and gender. The model was fitted with calf as a random intercept and age as a random slope. The error terms of the residuals were assumed to follow a normal distribution (mean of zero, variance of σ^2^) and to follow an autoregressive correlation structure of the first order. First-order interaction terms were tested and were retained if the interaction term was significant at a likelihood ratio test *p* value of 0.05 or less. Explanatory variables were retained in the final model if they achieved statistical significance at a likelihood ratio test *p* value of 0.05 or less. Overall model fit was based on the Akaike information criterion (AIC) and visual assessment of *Pearson’s* residuals against fitted values, and *Q-Q* standardised residuals against standardised normal quantiles were used to assess normality assumption. All analyses were conducted using nlme and lme4 [43] statistical packages in R.

## 3. Results

### 3.1. Live Weight Comparisons

The live weight measurements for the CON and TRT groups followed a similar trend (Table S1 and Figure S3). Calves in the TRT group had greater live weights (mean = 75 kg; standard deviation [SD] = 10 kg, *p* = 0.02) at weaning (Day 56) compared with calves in the CON group (mean = 69 kg, SD = 13 kg). Calves in the TRT group showed a tendency to be heavier on Day 42 (mean = 61 kg; SD = 8 kg; *p* = 0.09) compared with calves in the CON group (mean = 57 kg; SD = 11 kg). The average live weight gain, average daily live weight gain, average and total feed intake, and average feed efficiency were not statistically different between the groups.

### 3.2. Sequencing Results

The sequencing of faecal samples generated a total of 15,585,062 reads, of which 4,502,360 effective reads were ultimately analysed by the SILVA classifier after exclusion due to trimming and quality control. The number of effective merged reads per sample ranged from 7278 to 13,221 (median 9608; mean 11,032). Of these, 58% of the sequences could be assigned to the levels of phyla, class, order, and family, and 38% of the sequences could be assigned to the level of genus.

**Figure 1:**
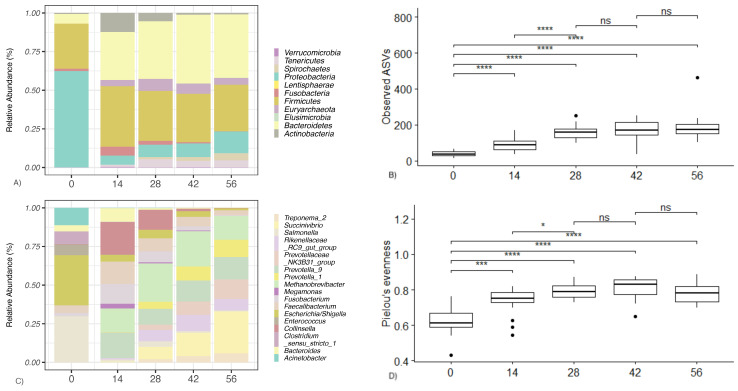
Changes in relative abundance of ASVs at the phyla (**A**) and genus level (**C**) in faecal samples from CON and TRT Groups at Days 0 (Birth), 14, 28, 42, and 56. Box and whisker plots showing level of microbial diversity (**B**) and evenness (**D**) of the calf faecal microbiome over time. Boxplots display the median as the middle line, whilst the perimeters of the box display the 1st and 3rd quantiles of the data. The whiskers extend to the highest and lowest values. ns not significant * *p* < 0.05 *** *p* < 0.01 **** *p* < 0.001. Solid black circles are potential outliers.

The phylogenetic classification demonstrated that bacterial communities were composed of 10 phyla, which were dominated by *Bacteroidetes* (average relative abundance = 32%), *Firmicutes* (32%), *Proteobacteria* (21%), *Euryarchaeota* (5%), *Actinobacteria* (4%), and *Tenericutes* (3%) (Figure 1A). *Seventeen* bacterial genera were most abundant over time (Figure 1C). The different bacterial genera were dominated by *Methanobrevibacter* (16%), *Prevotella_9* and *Succinivibrio* (11% each), *Escherichia*/*Shigella* (10%), *Faecalibacterium* (7%), *Collinsella* (7%), and *Salmonella* (7%). The phyla distribution for the CON and TRT calves followed a similar pattern, with the relative abundances for the two groups (CON, TRT) being as follows: *Bacteroidetes* (33%, 32%), *Firmicutes* (33%, 32%), *Proteobacteria* (18%, 22%), *Euryarchaeota* (5%, 4%), *Actinobacteria* (4%, 5%), and *Tenericutes* (3%, 3%).

At Day 0, the faecal microbiome was dominated by *Gammaproteobacteria* (mean 62%) and *Clostridia* (mean 19%). The abundances of *Bacteroidia*, *Fusobacteriia*, and *Coriobacteriia* were substantially lower (mean 6%, 2%, and 0.2%, respectively). By Day 14, however, the levels of *Gammaproteobacteria*, exclusively represented by *Enterobacteriaceae*, had declined to a steady-state abundance from Day 14 onwards (mean 7–13%). *Bacteroidia* (composed predominantly of *Bacteriodaceae*) displayed an opposite profile, exhibiting a lower abundance at Day 0 (6%), before rapidly increasing to mean abundances of 31%, 37%, 44%, and 41% at the subsequent sampling points. This was attributable to the emergence of *Prevotellaceae*, *Bacteroidaceae*, *Rikenellaceae,* and *Paraprevotellaceae*. The *Bacilli* abundance decreased to <1% by Day 42, due to decline in *Streptococcaceae*. *Actinobacteria*, with a mean abundance of <1% at Day 0, peaked at 3% on Day 14 (due to increased *Coriobacteriaceae*), before returning to a mean of 1% on Day 28. The mean levels of *Clostridia* remained stable between 3% and 5% of the total throughout the experiment. However, during successive time points, there was a marked decline in the abundance of *Clostridiaceae* and an increased abundance of *Prevotellaceae*, *Ruminococcaceae*, *Lachnospiraceae*, and *Muribaculaceae*.

In all calves, compared to Day 0, the microbial diversity and evenness showed a curvilinear increase between each sampling time point. The increase was linear between Day 0 and Day 28 (*p* < 0.05; Figure 1B; Data S1). The levels of microbial diversity did not differ after 28 days, whilst the level of microbial evenness plateaued between Day 28 and Day 42 and significantly decreased at Day 56 (*p* < 0.05). Both microbial diversity and evenness showed a significant curvilinear association with time in all calves, indicating an increase in the overall microbial diversity, with a gradual decrease after Day 28. This enhancement and changes in diversity were also demonstrated by the increasing numbers of distinct taxa at each time point. The faecal microbiome on Day 0 comprised an average of 131 ASVs, and it contained an average of 147 ASVs on Day 56 (Figure 1B; Data S1).

**Figure 2:**
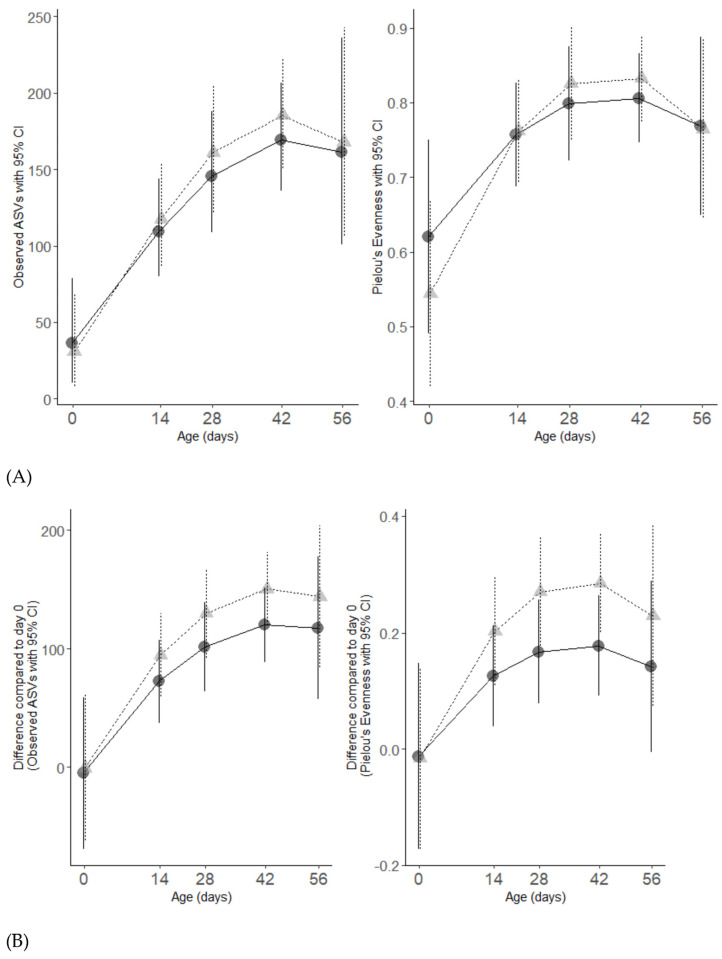
Line plots showing level of microbial diversity and evenness (**A**) and the difference in the level of microbial diversity (**B**) of the calf faecal microbiota over time for CON (solid grey triangles and dashed line) and TRT (solid black circle and solid black line) groups. Dashed and solid vertical lines display their 95% confidence intervals.

The absolute and relative change in the observed microbial alpha diversity and microbial evenness followed distinct curvilinear trends (*p* < 0.001). The alpha diversity and microbial evenness increased exponentially between Day 0 and Day 42 and decreased post Day 42. On average, when compared to the CON calves, the alpha diversity in the TRT calves was higher by Day 56 (*p* = 0.02), while no significant difference in microbial evenness was observed (Figure 2A). The rate of accumulation of the microbial diversity of the CON or TRT calves did not differ (Figure 2B). However, a difference in the rate of accumulation of evenness between the CON or TRT calves was observed. Compared to Day 0, the levels of microbial evenness (*p* = 0.02) increased at a faster rate in the CON calves compared with the TRT calves (Figure 1D). By comparing the change in microbial composition from Day 0 to each subsequent time point, on average, the TRT calves had a greater change in composition relative to Day 0 than the CON calves did (*p* = 0.02). This indicates that by Day 56, the composition of the microbiome in the TRT calves was significantly more dissimilar to Day 0 than it was in the CON calves (Figure 2B). This corresponds to the differences observed in alpha diversity, which demonstrated that DFM-fed calves accumulated a greater degree of diversity when compared to Day 0 than the CON calves did (Figure 2A,B).

**Figure 3:**
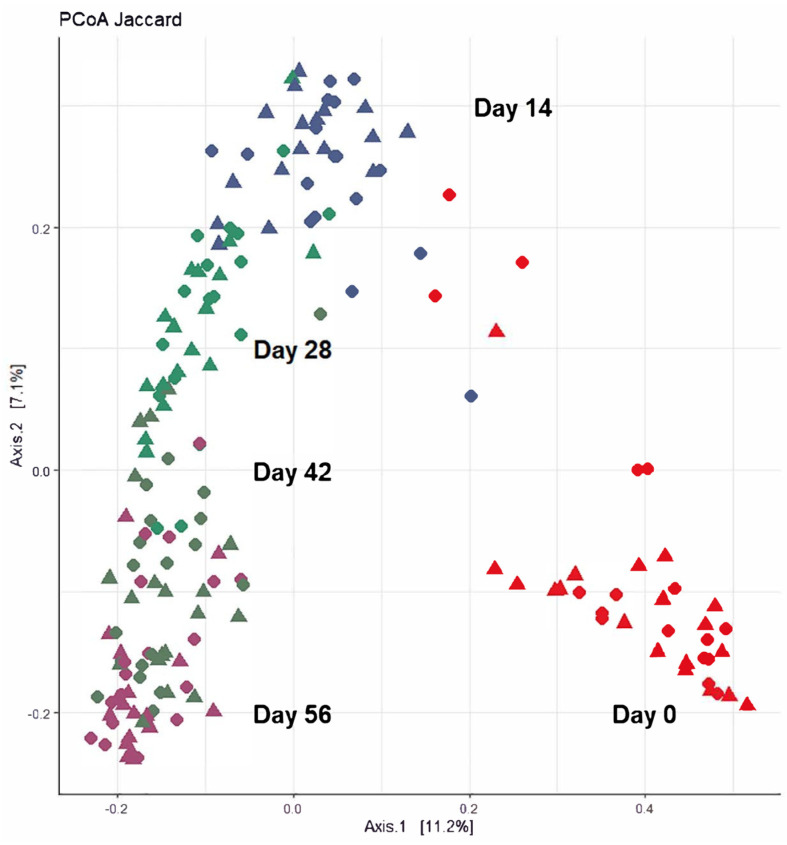
Clustering of calf faecal microbiome over time based on principal co-ordinates analysis (PCoA) of weighted unifrac using Bray–Curtis dissimilarity measures. Plotted using the first two principal co-ordinates, accounting for ~25% of the observed variation. Samples are coloured by individual sampling days, where solid triangles represent CON group and solid circles represent TRT group.

Moreover, the level of microbial dissimilarity was positively correlated with time, indicating that the microbial composition became significantly more divergent throughout the study period (PERMANOVA; *p* < 0.001). A strong correlation in microbial compositional abundance was explained with qualitative (unweighted UniFrac; rho = 0.62; *p* < 0.001) and quantitative dissimilarity measures (weighted UniFrac; rho = 0.51; *p* < 0.001), showing that compositional changes over time were related to both changes in the abundance of existing community members and the introduction of new species into the community. Furthermore, principal co-ordinates analysis (PCoA) of the *Jaccard* dissimilarity revealed that the faecal microbiota of all calves grouped into three distinct clusters: Day 0 (cluster 1), Days 14 and 28 (cluster 2), and Days 42 and 56 (cluster 3). This demonstrated that samples within each of these three clusters showed similar microbial community composition, but also that there was divergence in the microbial communities from the previous time period (Figure 3).

### 3.3. Compositional Differences between Different Groups

**Figure 4:**
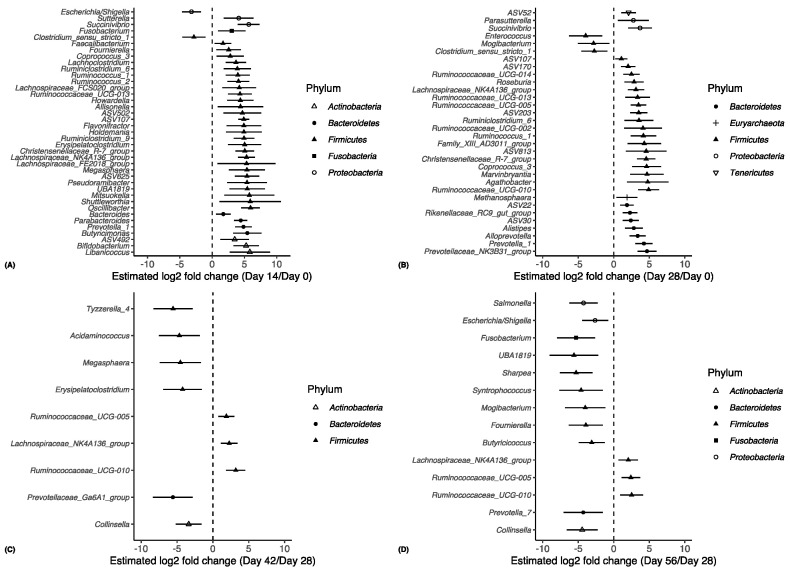
Significant log2-fold differences in bacterial abundance between calves’ age groups are presented. Bacterial abundances on Day 14 (**A**) and Day 28 (**B**) were compared to Day 0, and those at Day 42 (**C**) and Day 56 (**D**) were compared to Day 28. Each point is shaped according to its assigned phyla and represents a bacterial genus that showed a significant difference in abundance between the age groups compared. For example, in (**A**), a negative value denotes a log2-fold reduction in that genus on Day 14 compared to Day 0. Vertical dashed lines represent the null. Adjusted *p*-values of 0.01 or less were considered significant.

**Figure 5:**
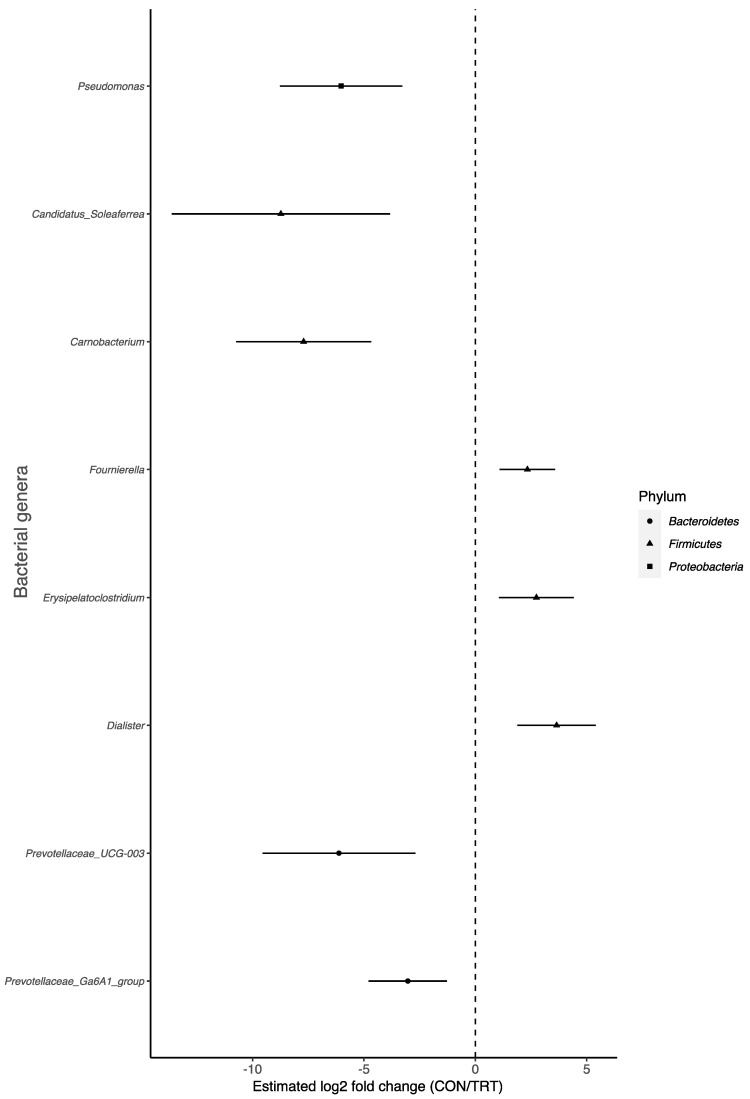
Significant log2-fold differences in bacterial families’ abundance between CON and TRT groups. Each point is shaped according to its assigned phyla and represents a bacterial genus that showed a significant difference in abundance. Negative values denote a log2-fold reduction in that genus in the CON compared to the TRT. Vertical dashed line represent the null. Adjusted *p*-values of 0.01 or less were considered significant.

To identify differences in the microbial composition of faecal samples between different groups, LEfSe was used to provide biomarkers at the genus level. Overall, there were 105 genera that were variably enriched in the CON and TRT groups on Days 0, 14, 28, 42, and 56 (Figure 4; Data S1). The relative abundance of eight genera differed (*p* < 0.001) between the CON and TRT groups. *Fournierella*, *Barnesiella*, and *Dialister* were significantly enriched in the CON group. *Carnobacterium*, *Pseudomonas*, *Prevotellaceae* UCG 003, *Prevotellaceae* Ga6A1 group, and *Candidatus_soleaferrea* were significantly enriched in the TRT group. The comparison between groups showed that *Bacteroidetes* were more prevalent in the TRT group, while *Firmicutes* were more prevalent in the CON group (Figure 5).

### 3.4. Metabolic Functions and Capacity of Ruminal and Faecal Microbiomes

**Figure 6:**
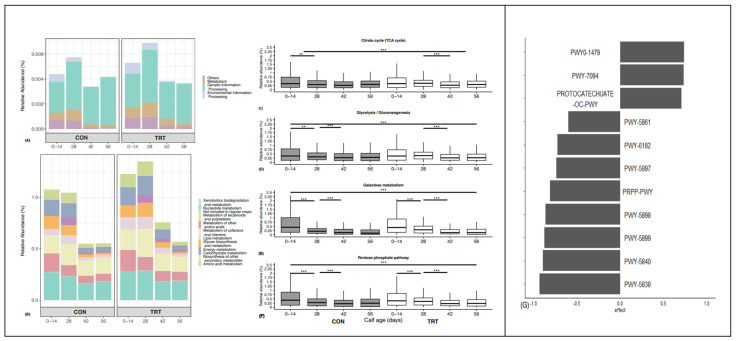
PICRUSt predicted summary of COG categories from 16S amplicon sequencing of faecal microbiomes. Relative abundances of level 1 (**A**) and level 2 metabolism (**B**) KEGG pathways are depicted by calf age within CON and TRT in barplots. Relative abundances of the four most abundant L3 KEGG pathways involved in carbohydrate metabolism (citrate cycle (**C**), glycolysis/gluconeogenesis (**D**), galactose metabolism (**E**), and pentose phosphate pathway (**F**)) at different ages (0–14, 28, 42, 56) for each group are presented in boxplots. Predicted functional pathways at Day 14 are shown in (**G**). ** *p* < 0.01, and *** *p* < 0.001 for the shown comparisons of L3 pathways.

To assess the metabolic potentials of faecal microbiomes, taxa were entered into PICRUSt2, and the inferred gene families were annotated against KOs and then collapsed into KEGG pathways. The gene families on Day 0 and Day 14 were collapsed into one category and used as the baseline. The gene families that were annotated to bacterial metabolism (Figure 6A) and carbohydrate metabolism (Figure 6B) declined (*p* < 0.01) post Day 28 (Figure 6A). The relative abundance of KEGG L2 pathways associated with carbohydrate metabolism did not differ (*p* > 0.05) at Day 42 and 56 but were both lower (*p* < 0.01) compared with the baseline and Day 28 (Figure 6B). A total of 288 KEGG level 3 pathways were identified, and 135 were related to carbohydrate metabolism. Of the microbiota-inferred gene families that were assigned to KEGG pathways, the four most abundant related to carbohydrate metabolism that showed variability (*p* < 0.01) between the CON and TRT groups were citrate cycle (TCA cycle; hsa00020), glycolysis/gluconeogenesis (hsa00010), galactose metabolism (hsa00052), and the pentose phosphate pathway (hsa00040; Figure 6C–F). For both the CON and TRT groups, galactose metabolism and the pentose phosphate pathway declined (*p* < 0.001) at a steady rate until Day 42. The glycolysis/gluconeogenesis pathway followed a similar trend for the CON but was stable in the TRT group (*p* > 0.05) for the first 28 days on DFM. The citrate cycle also followed the same trend. The functional pathway-level (*MetaCyc* pathway) predictions based on the metagenomics of gene families, weighted by the relative abundance of taxa, were explored for each time point and compared between the CON and TRT groups (Figure 6G). A total of 11 functional pathways were identified on Day 14 as contributing to the microbiota-wide pathway abundance (Benjamini–Hochberg [wi.eBH]-corrected *p* < 0.05). The expressions of eight functional pathways—predominantly menaquinol biosynthesis (PWY-5838, PWY-5840, PWY-5897/8/9), followed by demethylmenaquinol-8 biosynthesis (PWY-5861) and histidine, purine, and pyrimidine biosynthesis (PRPP-PWY)—were suppressed in the TRT group compared with the CON group. Conversely, fatty acid salvage (PWY-7094), tRNA processing (PWY0-1479), and ortho-cleavage pathway (Protocatechuate-ortho-cleavage-pwy) expressions were more pronounced in the CON compared to the TRT group (Figure 6F).

### 3.5. Gross Pathology and Histopathology Examination

The calves’ attributes and study measurement characteristics at postmortem are summarised in Tables S2–S7. Within the study calves, the LW and ADG did not differ (Table S2), and no gross pathological abnormalities were observed. Compared to calves in the CON group, calves in the TRT group had greater duodenum (with digesta) weights (mean = 87 g, SD = 64 g), greater abomasum (without digesta) weights (mean = 450 g, SD = 53 g), and greater reticulum (without digesta) weights (mean = 357 g, SD = 47 g) compared with calves in the CON group (mean = 33 g, SD = 5 g, *p* = 0.04; mean = 390 g, SD = 17 g, *p* = 0.05; mean = 257 g, SD = 31 g, [Bonferroni-adjusted] *p* = 0.05, respectively; Table S3). Rumen and intestinal development adaptation were more pronounced in the TRT calves (Tables S4–S7). On average, rumen and intestinal organs’ folding and crypts in the TRT calves were greater in length (mean = 1.8 mm, *p* = 0.03) and denser (2.1 unit, *p* = 0.09) than those of the CON calves (1.3 mm vs. 1.5 mm; Table S5). The calves in the TRT group had longer villi on the rumen ventral sac (TRT = 1.75 mm vs. CON = 1.31 mm, *p* = 0.04) and ileum (DFM = 0.80 mm vs. CON 0.62 mm, *p* < 0.01; Table S6). The TRT group’s calves’ omasum (DFM = 0.42 vs. CON = 0.66, *p* = 0.02) and colon (TRT = 0.09 vs. CON = 0.07, *p* = 0.02) villi widths were greater than those of the CON group (Table S6). The rumen’s ventral sac villi width (TRT = 0.37 mm vs. CON = 0.22 mm, *p* = 0.06), caecum (TRT = 0.07 vs. CON = 0.05, *p* = 0.07) villi width, surface area of the duodenum (TRT = 0.50 μm vs. CON = 0.38 μm, *p* = 0.08), and middle jejunum (TRT = 0.42 vs. CON = 0.50, *p* = 0.09) tended to be greater in the TRT group compared with the CON (Table S7).

## 4. Discussion

Early-life gastrointestinal microbial communities critically influence the growth, development, and immunity of the host [6,44]. Understanding the colonisation and succession patterns of the gut microbiota provides crucial insights into host–microbiome interactions during the post-natal phase. In the current study, we examined faecal samples of the study animals. The composition and function of the faecal microbiota are representative of the lower gut and were found to be diverse and age-dependent throughout the post-natal period, which is similar to findings reported in previous studies [6,10,45]. Changes in the microbiota structure and function are likely to be in response to the nutrient availability in the gut and could have assisted in GIT development and in the transition process from liquid to solid throughout the post-natal period until weaning [6,46].

We show here that from the first few weeks of life until weaning, there is rapid evolution and emergence of diverse species in the neonatal calf’s gut microbiome. Across all faecal samples, little difference was observed between the groups in the most abundant phyla (Figure 1). Our results show that the intestinal microbiota at birth is characterised by low diversity and a relative dominance of three phyla: *Proteobacteria*, *Firmicutes,* and *Bacteroidetes*. This is in agreement with other studies in which these phyla were the most abundant in this age group [7,13,47]. The first facultative anaerobe colonisers aid the establishment of other bacterial groups, most notably *Methanobrevibacter*, *Prevotella_9*, *Succinivibrio*, and *Escherichia*/*Shigella*, by maintaining enteric anoxia [6,45,48]. There was numerical difference between the two groups in the most abundant phyla, notably *Proteobacteria* (a reduction of 4% in favour of the TRT group). Whilst this dominance could be considered a sign of dysbiosis [49,50,51], members of the *Proteobacteria* decline rapidly, and the arrival and dominance of *Firmicutes* and *Bacteroidetes* increase over time.

In the current study, calves remained healthy and did not become clinically ill during the study period. The supplementation of DFM in calves may yield the greatest advantages during periods of health challenges [52,53]. Both groups of calves in this study were pre-screened as healthy, housed in separate individual pens, and their care followed strict animal husbandry and biosecurity practices, all of which could have contributed significantly to the absence of illness. Calf’s live weight at weaning (Day 56) was higher in the treatment group, in agreement with previous reports [45,48,54,55,56]. However, no difference in live weight was observed prior to Day 42, and differences in ADG or dry matter intake were not detected between groups. As calves grew older, those in the treatment group had a higher relative change in microbial richness and evenness indices over time compared with the control calves. These findings indicate that distinctive changes at the species level could explain differences in the live weight gains that were observed as the animal transitioned closer toward a solid feed ration [18,45,48,57]. *Carnobacterium* spp. were amongst the bacterial classes that were over-represented in the TRT group. Both *Carnobacterium* spp. and the DFM organisms are known as bacteriocin-producing bacteria that exhibit a wide spectrum of activity against known gastrointestinal pathogens such as *Listeria monocytogenes* [58,59,60]. As calves transition to a solid diet, the challenge to the GIT tract from exposure to the pathogens increases and puts pressure on the immune system, which in turn demands a diversion of energy and other metabolic resources. The proliferation of bacteria that are capable of producing bacteriocins increases the likelihood of inhibitory actions against GIT pathogens and spares the metabolic resource loss for growth and ongoing maturation. Further in vivo and in vitro work is required to verify these findings.

Moreover, rumen and intestinal organs’ folding, crypts, papillae length, and intestinal villi density and villi length were more pronounced in the TRT calves and could have also contributed to the observed differences in weight gain at weaning. Ruminal papillae are critical in nutrient absorption [61]. The TRT calves in this study had an increased nutrient-absorptive area of their ruminal papillae compared to the control animals. The papillae growth in the DFM-treated calves in the present study could be related to increased short chain fatty acids (SCFAs) (propionate, butyrate; [52]), because SCFAs are a by-product of microbial fermentation and a healthy rumen epithelium. The observed difference in papillary growth supports the suggested study finding that the rumen is more developed in the DFM-treated calves. The effect of the DFM may have also extended to the small intestine and hind gut. In the ileum, the total height, villus height, and crypt depth were greater for the TRT calves at weaning compared with the control group. A portion of the DFM may have bypassed the developing forestomach and colonised, or otherwise influenced, the structure and perhaps function of the ileum. Taller villi, akin to papillary growth in this study, may signify an enhanced absorptive area for improved nutrient uptake within the intestinal tract.

*Fournierella* spp. and *Dialister* spp. were enriched in the CON calves. They have been found to interfere with the metabolism of amino acids and glucose in gastrointestinal disorders [62,63,64]. This, in turn, reduces the availability of nutrients that are necessary for growth and development. The predicted metagenomes (PICRUSt) of rumen and faeces microbiomes confirmed those findings and showed a significant reduction in genes associated with carbohydrate metabolism between the two groups. In agreement with previous studies [14,65], this suggests that DFM supplementation initiated a shift in the microbiome structure and function towards that of the mature rumen at an earlier point in post-natal life (approx. 6 weeks of age) compared with the control group. However, caution should be exercised when interpreting these findings. PICRUSt are dynamic predictions that depend on discerned functions within microbial communities residing in the gastrointestinal tract of humans and animals, and they are derived from comprehensive whole-genome shotgun sequencing of samples. Given the paucity of shotgun sequencing studies in ruminants, the precision of PICRUSt in gauging the functionality of the ruminant gastrointestinal microbiota may be subject to over- or under-estimation.

The results of the current study should be interpreted with care, as there is strong internal validity with limited external validity. Therefore, the findings of the current study can only be extrapolated to similar cattle populations that have also been similarly managed. In addition, the animal sample size calculation was not optimised towards microbiota analyses. Therefore, the sample size is considered small, and the study is likely to be under-powered. Larger cohorts should be evaluated in future studies. Finally, we did not incorporate biomarkers or perform microbiota analyses by other methods. Thus, it is not possible to determine the precise mechanism(s) of action or how the microbiota results correlate with the observed phenotypic and histology changes in the gastrointestinal tract. Future studies should assess these changes in a longitudinal study and work towards identifying biomarkers of interest as the animals transition onto solid feed rations. These future studies should also expand analyses such as host responses and transcriptomics to more fully understand the mechanisms by which a host’s intestinal mucosa and the DFM interact.

## 5. Conclusions

Supplementing calves with the LAB-based DFM increased the live weight at weaning and influenced the microbiota diversity and evenness of the intestinal tract. Calves in the TRT group had greater duodenum, abomasum, and reticulum weights, as well as more pronounced rumen and intestinal development. The relative abundance of eight bacterial genera differed between the treatment groups. Future work is needed to better understand the potential association of DFM products with gut mucosa-associated microbiota.

## Data Availability

Data available on request from first author.

## Supplementary Materials

The following supporting information can be downloaded at https://www.mdpi.com/article/10.3390/ani14050693/s1: Figure S1: Caecum histology samples with an example of area selection for linear measurement; forestomach (reticulum, rumen, omasum) linear measurements were obtained through measurement of all intact villi to ensure that the minimum sample number needed for significance was measured (See Figure S2). To obtain an adequate number of measurements, histological recuts were acquired and measured; Figure S2: Linear measurements of forestomach example; Table S1: Descriptive statistics of live weight (kg), live weight gain (kg), average daily gain (g/d), average daily feed intake (kg/d), total feed intake (kg), and feed efficiency by experimental group (Control—CON—and Treatment—TRT). Table S2: Descriptive statistics of live weight, feed intake and body measurements of animals randomly selected from each experimental group (Control—CON—and Treatment—TRT) for postmortem and histopathology investigation; Table S3: Descriptive statistics of gastrointestinal tracts’ organ weights (g) of animals randomly selected from each experimental group (Control—CON—and Treatment—TRT) for postmortem and histopathology investigation; Table S4: Descriptive statistics of gastrointestinal tracts’ organ lengths (mm) of animals randomly selected from each experimental group (Control—CON—and Treatment—TRT) for postmortem and histopathology investigation; Table S5: Descriptive statistics of organ tissue’s fold density and length of animals randomly selected from each experimental group (Control—CON—and Treatment—TRT) for postmortem and histopathology investigation; Table S6: Organ histopathology microscopic villi length (mm) and width (mm) and histopathological measurements of animals randomly selected from Control (CON) and Treatment (TRT) for postmortem and histopathology investigation; Table S7: Organ histopathology microscopic villi surface area (μm) of animals randomly selected from each experimental group (Control—CON—and Treatment—TRT) for postmortem and histopathology investigation; Data S1: Significant log2-fold differences in bacterial abundance between calves’ age groups are presented. Bacterial abundances on Day 14 (A) and Day 28 (B) were compared to Day 0, and those at Day 42 (C) and Day 56 (D) were compared to Day 28. Each point is shaped according to its assigned phyla and represents a bacterial genus that showed a significant difference in abundance between the age groups compared. For example, in A, a negative value denotes a log2-fold reduction in that genus on Day 14 compared to Day 0. Adjusted *p*-values of 0.01 or less were considered significant.

## References

[1] Du W., Wang X., Hu M., Hou J., Du Y., Si W., Yang L., Xu L., Xu Q. (2023). Modulating gastrointestinal microbiota to alleviate diarrhea in calves. Front. Microbiol..

[2] Zhao Q., Elson C.O. (2018). Adaptive immune education by gut microbiota antigens. Immunology.

[3] Fan P., Bian B., Teng L., Nelson C.D., Driver J., Elzo M.A., Jeong K.C. (2020). Host genetic effects upon the early gut microbiota in a bovine model with graduated spectrum of genetic variation. ISME J..

[4] Torow N., Hornef M.W. (2017). The neonatal window of opportunity: Setting the stage for life-long host-microbial interaction and immune homeostasis. J. Immunol..

[5] Gensollen T., Iyer S.S., Kasper D.L., Blumberg R.S. (2016). How colonization by microbiota in early life shapes the immune system. Science.

[6] Amin N., Schwarzkopf S., Troscher-Mussotter J., Camarinha-Silva A., Danicke S., Huber K., Frahm J., Seifert J. (2023). Host metabolome and faecal microbiome shows potential interactions impacted by age and weaning times in calves. Anim. Microbiome.

[7] Alipour M.J., Jalanka J., Pessa-Morikawa T., Kokkonen T., Satokari R., Hynonen U., Iivanainen A., Niku M. (2018). The composition of the perinatal intestinal microbiota in cattle. Sci. Rep..

[8] Dias J., Marcondes M.I., Motta de Souza S., Cardoso Da Mata E Silva B., Fontes Noronha M., Tassinari Resende R., Machado F.S., Cuquetto Mantovani H., Dill-Mcfarland K.A., Suen G. (2018). Bacterial community dynamics across the gastrointestinal tracts of dairy calves during preweaning development. Appl. Environ. Microbiol..

[9] Svetlana Ferreira L., de Sousa Bicalho M., Bicalho R.C. (2019). The *Bos taurus* maternal microbiome: Role in determining the progeny early-life upper respiratory tract microbiome and health. PLoS ONE.

[10] Badman J., Daly K., Kelly J., Moran A.W., Cameron J., Watson I., Newbold J., Shirazi-Beechey S.P. (2019). The effect of milk replacer composition on the intestinal microbiota of pre-ruminant dairy calves. Front. Vet. Sci..

[11] Robertson R.C., Manges A.R., Finlay B.B., Prendergast A.J. (2019). The Human microbiome and child growth-first 1000 days and beyond. Trends Microbiol..

[12] Guilloteau P., Zabielski R., David J.C., Blum J.W., Morisset J.A., Biernat M., Wolinski J., Laubitz D., Hamon Y. (2009). Sodium-butyrate as a growth promoter in milk replacer formula for young calves. J. Dairy Sci..

[13] Malmuthuge N., Griebel P.J., Guan L.L. (2015). The gut microbiome and its potential role in the development and function of newborn calf gastrointestinal tract. Front. Vet. Sci..

[14] Meale S.J., Li S.C., Azevedo P., Derakhshani H., DeVries T.J., Plaizier J.C., Steele M.A., Khafipour E. (2017). Weaning age influences the severity of gastrointestinal microbiome shifts in dairy calves. Sci. Rep..

[15] Bladwin VI R.L., McLeod K.R., Klotz J.L., Heitmann R.N. (2004). Rumen development, intestinal growth and hepatic metabolism in the pre- and postweaning ruminant. J. Dairy Sci..

[16] Cezar A.M., Donde S.C., Tomaluski C.R., da Silva A.P., Toledo A.F., Coelho M.G., Virginio Junior G.F., Bittar C.M.M. (2022). Age and post-prandial variations on selected metabolites in dairy calves fed different liquid diets. Animals.

[17] Abecia L., Martin-Garcia A.I., Martinez G., Newbold C.J., Yanez-Ruiz D.R. (2013). Nutritional intervention in early life to manipulate rumen microbial colonization and methane output by kid goats postweaning. J. Anim. Sci..

[18] Meale S.J., Popova M., Saro C., Martin C., Bernard A., Lagree M., Yanez-Ruiz D.R., Boudra H., Duval S., Morgavi D.P. (2021). Early life dietary intervention in dairy calves results in a long-term reduction in methane emissions. Sci. Rep..

[19] Jiao J., Wu J., Zhou C., Tang S., Wang M., Tan Z. (2016). Composition of ileal bacterial community in grazing goats varies across non-rumination, transition and rumination stages of life. Front. Microbiol..

[20] Walter J. (2008). Ecological role of lactobacilli in the gastrointestinal tract: Implications for fundamental and biomedical research. Appl. Environ. Microbiol..

[21] Du R., Jiao S., Dai Y., An J., Lv J., Yan X., Wang J., Han B. (2018). Probiotic *Bacillus amyloliquefaciens* C-1 improves growth performance, stimulates gh/igf-1, and regulates the gut microbiota of growth-retarded beef calves. Front. Microbiol..

[22] Kelly A., McCabe M., Kenny D., Waters S. (2018). Effect of a butyrate-fortified milk replacer on gastrointestinal microbiota and products of fermentation in artificially reared dairy calves at weaning. Sci. Rep..

[23] Schofield B.J., Lachner N., Le O.T., McNeill D.M., Dart P., Ouwerkerk D., Hugenholtz P., Klieve A.V. (2018). Beneficial changes in rumen bacterial community profile in sheep and dairy calves as a result of feeding the probiotic *Bacillus amyloliquefaciens* H57. J. Appl. Microbiol..

[24] Minato H., Otsuka M., Shirasaka S., Itabashi H., Mitsumori M. (1992). Colonization of microorganisms in the rumen of young calves. J. Gen. Appl. Microbiol..

[25] Barreto M.O., Soust M., Moore R.J., Olchowy T.W.J., Alawneh J.I. (2021). Systematic review and meta-analysis of probiotic use on inflammatory biomarkers and disease prevention in cattle. Prev. Vet. Med..

[26] Virginio Junior G.F., Bittar C.M.M. (2021). Microbial colonization of the gastrointestinal tract of dairy calves—A review of its importance and relationship to health and performance. Anim. Health Res. Rev..

[27] Mansilla F.I., Ficoseco C.A., Miranda M.H., Puglisi E., Nader-Macias M.E.F., Vignolo G.M., Fontana C.A. (2022). Administration of probiotic lactic acid bacteria to modulate fecal microbiome in feedlot cattle. Sci. Rep..

[28] Hewitt A., Olchowy T., James A.S., Fraser B., Ranjbar S., Soust M., Alawneh J.I. (2020). Linear body measurements and productivity of subtropical Holstein-Friesian dairy calves. Aust. Vet. J..

[29] Bielmann V., Gillan J., Perkins N.R., Skidmore A.L., Godden S., Leslie K.E. (2010). An evaluation of Brix refractometry instruments for measurement of colostrum quality in dairy cattle. J. Dairy Sci..

[30] Weaver D.M., Tyler J.W., VanMetre D.C., Hostetler D.E., Barrington G.M. (2000). Passive transfer of colostral immunoglobulins in calves. J. Vet. Int. Med..

[31] Chigerwe M., Tyler J.W., Schultz L.G., Middleton J.R., Steevens B.J., Spain J.N. (2008). Effect of colostrum administration by use of oroesophageal intubation on serum IgG concentrations in Holstein bull calves. Am. J. Vet. Res..

[32] Engelbrektson A., Kunin V., Wrighton K.C., Zvenigorodsky N., Chen F., Ochman H., Hugenholtz P. (2010). Experimental factors affecting PCR-based estimates of microbial species richness and evenness. ISME J..

[33] Callahan B.J., McMurdie P.J., Rosen M.J., Han A.W., Johnson A.J., Holmes S.P. (2016). DADA2: High-resolution sample inference from Illumina amplicon data. Nat. Methods.

[34] Price M.N., Dehal P.S., Arkin A.P. (2010). FastTree 2--approximately maximum-likelihood trees for large alignments. PLoS ONE.

[35] Douglas G.M., Maffei V.J., Zaneveld J.R., Yurgel S.N., Brown J.R., Taylor C.M., Huttenhower C., Langille M.G.I. (2020). PICRUSt2 for prediction of metagenome functions. Nat. Biotechnol..

[36] Fernandes A.D., Reid J.N., Macklaim J.M., McMurrough T.A., Edgell D.R., Gloor G.B. (2014). Unifying the analysis of high-throughput sequencing datasets: Characterizing RNA-seq, 16S rRNA gene sequencing and selective growth experiments by compositional data analysis. Microbiome.

[37] Fernandes A.D., Macklaim J.M., Linn T.G., Reid G., Gloor G.B. (2013). ANOVA-Like differential gene expression analysis of single-organism and meta-RNA-Seq. PLoS ONE.

[38] R Development Core Team (2012). R: A Language and Environment for Statistical Computing.

[39] Kanehisa M., Goto S. (2000). KEGG: Kyoto encyclopedia of genes and genomes. Nucleic Acids Res..

[40] Du J., Yuan Z., Ma Z., Song J., Xie X., Chen Y. (2014). KEGG-PATH: Kyoto encyclopedia of genes and genomes-based pathway analysis using a path analysis model. Mol. Biosyst..

[41] Dohoo I.R., Martin W., Stryhn W. (2009). Veterinary Epidemiologic Research.

[42] Stevenson M., Nunes T., Sanchez J., Thornton R. epiR: Functions for Analysing Epidemiological Data. R Package, Version 0.9-3; 2008. Epi Center, Massey University, New Zealand. http://epicentre.massey.ac.nz.

[43] Bates D. (2007). lme4: Linear Mixed-Effects Models Using S4 Classes. R Package, Version 0.99875-9. https://cran.r-project.org/web/packages/lme4/lme4.pdf.

[44] Kenez A., Koch C., Korst M., Kesser J., Eder K., Sauerwein H., Huber K. (2018). Different milk feeding intensities during the first 4 weeks of rearing dairy calves: Part 3: Plasma metabolomics analysis reveals long-term metabolic imprinting in Holstein heifers. J. Dairy Sci..

[45] Oikonomou G., Teixeira A.G.V., Foditsch C., Bicalho M.L., Machado V.S., Bicalho R.C. (2013). Fecal microbial diversity in pre-weaned dairy calves as described by pyrosequencing of metagenomic 16S rDNA. Associations of *Faecalibacterium* species with health and growth. PLoS ONE.

[46] Schwarzkopf S., Kinoshita A., Kluess J., Kersten S., Meyer U., Huber K., Dänicke S., Frahm J. (2019). Weaning holstein calves at 17 weeks of age enables smooth transition from liquid to solid feed. Animals.

[47] Malmuthuge N., Griebel P.J., le Guan L. (2014). Taxonomic identification of commensal bacteria associated with the mucosa and digesta throughout the gastrointestinal tracts of preweaned calves. Appl. Environ. Microbiol..

[48] Kim E.T., Lee S.J., Kim T.Y., Lee H.G., Atikur R.M., Gu B.H., Kim D.H., Park B.Y., Son J.K., Kim M.H. (2021). Dynamic changes in fecal microbial communities of neonatal dairy calves by aging and diarrhea. Animals.

[49] Hennessy M., Indugu N., Vecchiarelli B., Redding L., Bender J., Pappalardo C., Leibstein M., Toth J., Stefanovski D., Katepalli A. (2021). Short communication: Comparison of the fecal bacterial communities in diarrheic and nondiarrheic dairy calves from multiple farms in southeastern Pennsylvania. J. Dairy Sci..

[50] Maharshak N., Packey C.D., Ellermann M., Manick S., Siddle J.P., Huh E.Y., Plevy S., Sartor R.B., Carroll I.M. (2013). Altered enteric microbiota ecology in interleukin 10-deficient mice during development and progression of intestinal inflammation. Gut Microbes.

[51] Singh P., Teal T.K., Marsh T.L., Tiedje J.M., Mosci R., Jernigan K., Zell A., Newton D.W., Salimnia H., Lephart P. (2015). Intestinal microbial communities associated with acute enteric infections and disease recovery. Microbiome.

[52] Cappellozza B.I., Copani G., Boll E.J., Queiroz O. (2023). Supplementation of direct-fed microbial *Enterococcus faecium* 669 affects performance of preweaning dairy calves. JDS Commun..

[53] Roodposhti P.M., Dabiri N. (2012). Effects of probiotic and prebiotic on average daily gain, fecal shedding of *Escherichia Coli*, and immune system status in newborn female calves. Asian-Australas. J. Anim. Sci..

[54] Wang L., Sun H., Gao H., Xia Y., Zan L., Zhao C. (2023). A meta-analysis on the effects of probiotics on the performance of pre-weaning dairy calves. J. Anim. Sci. Biotechnol..

[55] Dick K.J., Duff G.C., Limesand S.W., Cuneo S.P., Knudson D.K., McMurphy C.P., Hall L.W., Bernal-Rigoli J.C., Marchello J.A. (2013). Effects of a direct-fed microbial on digestive-tract morphology of Holstein bull calves and performance and carcass characteristics of Holstein steers. Prof. Anim. Sci..

[56] Amin N., Seifert J. (2021). Dynamic progression of the calf’s microbiome and its influence on host health. Comput. Struct. Biotechnol. J..

[57] Dill-McFarland K.A., Breaker J.D., Suen G. (2017). Microbial succession in the gastrointestinal tract of dairy cows from 2 weeks to first lactation. Sci. Rep..

[58] Pilchova T., Pilet M.F., Cappelier J.M., Pazlarova J., Tresse O. (2016). Protective effect of *Carnobacterium* spp. against *Listeria monocytogenes* during host cell invasion using in vitro HT29 model. Front. Cell Infect. Microbiol..

[59] Józefiak D., Sip A., Kaczmarek S., Rutkowski A. (2010). The effects of *Carnobacterium divergens* AS7 bacteriocin on gastrointestinal microflora in vitro and on nutrient retention in broiler chickens. J. Anim. Feed. Sci..

[60] Pilet M.F., Dousset X., Barré R., Novel G., Desmazeaud M., Piard J.C. (1995). Evidence for two bacteriocins produced by *Carnobacterium pisciola* and *Carnobacterium divergens* isolated from fish and active against *Listeria monocytogenes*. J. Food Prot..

[61] Lesmeister K.E., Heinrichs A.J. (2004). Effects of corn processing on growth characteristics, rumen development, and rumen parameters in neonatal dairy calves. J. Dairy Sci..

[62] Yan Z., Zhang K., Zhang K., Wang G., Wang L., Zhang J., Qiu Z., Guo Z., Song X., Li J. (2022). Integrated 16S rDNA Gene sequencing and untargeted metabolomics analyses to investigate the gut microbial composition and plasma metabolic phenotype in calves with dampness-heat diarrhea. Front. Vet. Sci..

[63] Wright E.K., Kamm M.A., Teo S.M., Inouye M., Wagner J., Kirkwood C.D. (2015). Recent advances in characterizing the gastrointestinal microbiome in Crohn’s disease: A systematic review. Inflamm. Bowel Dis..

[64] An G., Zhang Y., Fan L., Chen J., Wei M., Li C., Chen X., Zhang L., Yang D., Wang J. (2021). Integrative analysis of vaginal microorganisms and serum metabolomics in rats with estrous cycle disorder induced by long-term heat exposure based on 16s rDNA gene sequencing and LC/MS-based metabolomics. Front. Cell Infect. Microbiol..

[65] Meale S.J., Li S., Azevedo P., Derakhshani H., Plaizier J.C., Khafipour E., Steele M.A. (2016). Development of ruminal and fecal microbiomes are affected by weaning but not weaning strategy in dairy calves. Front. Microbiol..

