# Supplementary material for: Effect of a Lactobacilli-Based Direct-Fed Microbial Product on Gut Microbiota and Gastrointestinal Morphological Changes
Source: Animals (Basel). 2024 Feb 23;14(5):693. doi: 10.3390/ani14050693 (PMC10931233; doi:10.3390/ani14050693)
Supplement: Supplementary file 1 [file animals-14-00693-s001.zip › animals-2875175-supplementary.pdf]

Table S1. Descriptive statistics of live weight (kg), live weight gain (kg), average daily gain (g/d), average daily feed intake (kg/d), total feed intake (kg), and feed efficiency by Experimental Group (Control – CON and Treatment -TRT).

		**Group CON**			**Group TRT**			**P value** F
	**Study Day**	**Mean (SD)**	**Median (Q1, Q3)**	**Min, Max**	**Mean (SD)**	**Median (Q1, Q3)**	**Min, Max**	
**Live weight (kg)**	-3	39.71 (6.97)	41.00 (35.30, 43.70)	22.20, 53.20	42.16 (6.26)	40.90 (38.70, 45.10)	30.60, 53.80	0.30
	14	42.73 (7.34)	42.00(38.2, 47.20)	25.00, 55.80	45.12 (6.37)	44.00 (41.85, 50.00)	36.20, 58.00	0.31
	28	48.73 (8.79)	48.20 (42.55, 53.95)	31.80, 65.20	52.04 (6.06)	51.40 (48.65, 53.70)	42.00, 64.80	0.18
	42	57.13 (10.58)	55.90 (49.20, 65.30)	37.00, 73.40	61.38 (8.42)	60.40 (56.80, 66.00)	42.400, 81.00	0.09
	56	69.18 (13.37)	68.90 (61.40, 79.30)	41.80, 91.60	75.01 (9.66)	74.10 (70.60, 77.80)	53.400, 101.00	0.02
**Average daily gain (g/d)**	-3 to 14	180 (150)	160 (120, 260)	-90, 520	170 (150)	180 (50, 280)	-110, 460	0.96
	15 to 28	430 (250)	450 (250, 620)	-70, 830	490 (190)	500 (370, 650)	130, 760	0.33
	29 to 42	600 (290)	690 (370, 830)	-10, 1010	670 (250)	680 (490, 850)	30, 1240	0.34
	43 to 56	860 (310)	870 (770, 1060)	170, 1440	970 (210)	960 (820, 1100)	600, 1430	0.11
	**-3 to 56**	500 (180)	520 (380, 620)	180, 840	570 (90)	540 (500, 610)	450, 820	0.14
**Total feed intake(kg)**	**-3 to 56**	42.73 (17.54)	42.84 (33.40, 51.00)	15.56, 88.30	46.59 (12.19)	45.60 (38.96, 52.40)	26.97, 70.44	0.34
**Feed efficiency §**	**-3 to 56**	1.46 (0.49)	1.34 (1.18, 1.48)	0.84, 3.18	1.44 (0.33)	1.44 (1.19, 1.70)	0.90, 2.00	0.62

Key: SD - Standard Deviation, Q1 & Q3 - First and Third Quartiles, Min - Minimum, Max - Maximum
§Average Feed intake kg : Average daily gain kg
F P values mean estimates. P values are interaction term p values derived from a mixed effects regression model of each measured outcome variable over the study period. Model fitted with calf as random effect with random intercepts and an autoregressive correlation structure for the residuals.

Table S2. Descriptive statistics of live weight, feed intake and body measurements of animals randomly selected from each Experimental Group (Control – CON and Treatment -TRT) for postmortem and histopathology investigation. .

Variable	Group CON (n = 3)			Group TRT (n = 3)			P values‡
	Mean (SD)	Median (Q1, Q3)	Min, Max	Mean (SD)	Median (Q1, Q3)	Min, Max	
**Age at post-mortem (days)**	63 (4)	64 (62, 65)	59, 66	62 (3)	61 (60, 63)	59, 65	0.66
**Initial live weight (kg)**	46 (7)	43 (42, 48)	41, 53	49 (5)	49 (47, 52)	44, 54	0.28
**Live weight at post-mortem (kg)**	77 (12)	73 (71, 82)	68, 91	84 (6)	85 (81, 87)	77, 89	0.51
**Total live weight gain (kg)**	32 (6)	30 (29, 34)	27, 38	35 (2)	36 (34, 36)	33, 36	0.51
**Average daily gain (kg/d)**	0.49 (0.86)	0.48 (0.47, 0.52)	0.46, 0.58	0.56 (0.79)	0.59 (0.57, 0.57)	0.55, 0.55	0.51
**Total feed intake (kg)** §§	51 (19)	41 (39, 57)	38, 73	51 (1)	51 (50, 52)	49, 52	0.51
**Hip Height (cm)**	93(2)	92 (91, 94)	91, 95	95 (2)	95 (94, 96)	93, 97	0.18
**Hip Width (cm)**	21 (1)	20 (20, 21)	20, 22	22 (1)	22 (21, 22)	21, 22	0.28
**Heart Girth (cm)**	103 (7)	105 (100, 107)	95, 109	102 (2)	103 (102, 104)	100, 104	0.51

Key: SD - Standard Deviation, Q1 & Q3 - First and Third Quartiles, Min - Minimum, Max - Maximum
§§ Feed intake from grain only.
‡ P values derived from *Kruskal-Wallis* chi-squared test and were adjusted (Bonferroni) for multiple comparisons. The null hypothesis is that data distribution is homogenous. P values ≤0.05 reject the null hypothesis.

Table S3. Descriptive statistics of gastrointestinal tracts organs weights (g) of animals randomly selected from each Experimental group (Control – CON and Treatment -TRT) for postmortem and histopathology investigation.

Organ	Group CON (n = 3)			Group TRT (n = 3)			P value ‡
	Mean (SD)	Median (Q1, Q3)	Min, Max	Mean (SD)	Median (Q1, Q3)	Min, Max	
**Rumen pH**	6 (0.89)	5.7 (5.5, 6.35)	5.3, 7.0	5.6 (0.1)	5.6 (5.55, 5.65)	5.5, 5.7	0.66
**Weight with digesta (g)**							
**Rumen**	8403 (1443)	8510 (7710, 9150)	6910, 9790	8370 (1164)	8690 (7885, 9015)	7080, 9340	0.82
**Abomasum**	1620 (652)	1940 (1405, 1995)	870, 2050	1390 (450)	1640 (1255, 1650)	870, 1660	0.38
**Omasum**	590 (291)	700 (480, 755)	260, 810	760 (221)	840 (675, 885)	510, 930	0.28
**Reticulum**	590 (324)	530 (415, 735)	300, 940	760 (243)	720 (630, 870)	540, 1020	0.28
**Duodenum**	33 (5)	30 (30, 35)	30, 40	87 (64)	50 (50, 105)	50, 160	0.04
**Jejunum**	3590 (922)	3220 (3065, 3930)	2910, 4640	4243.33 (134)	4300 (4195, 4320)	4090, 4340	0.51
**Ileum**	37 (15)	40 (30, 45)	20, 50	30 (17)	40 (25, 40)	10, 40	0.48

**Caecum**	730 (147)	810 (685, 815)	560, 820	607 (380)	730 (455, 820)	180, 910	0.83
**Colon**	1077 (352)	1030 (890, 1240)	750, 1450	1367 (279)	1310 (1215, 1490)	1120, 1670	0.28
**Weight without digesta (g)**							
**Rumen**	1460 (191)	1560 (1400, 1570)	1240, 1580	1427 (84)	1470 (1400, 1475)	1330, 1480	0.51
**Abomasum**	390 (17)	400 (385, 400)	370, 400	450 (53)	430 (420, 470)	410, 510	**0.05**
**Omasum**	413.33 (146)	460 (355, 495)	250, 530	417 (32)	430 (405, 435)	380, 440	0.51
**Reticulum**	257 (31)	250 (240, 270)	230, 290	357 (47)	340 (330, 375)	320, 410	**0.05**
**Duodenum**	33 (6)	30 (30, 35)	30, 40	73 (58)	40 (40, 90)	40, 140	0.09
**Jejunum**	2050 (579)	1930 (1735, 2305)	1540, 2680	2643 (464)	2540 (2390, 2845)	2240, 3150	0.27
**Ileum**	33 (12)	40 (30, 40)	20, 40	30 (17)	40 (25, 40)	10, 40	0.79
**Caecum**	227 (49)	250 (210, 255)	170, 260	197 (97)	220 (155, 250)	90, 280	0.82
**Colon**	510 (132)	560 (460, 585)	360, 610	677 (84)	650 (630, 710)	610, 770	0.07

Key: SD - Standard Deviation, Q1 & Q3 - First and Third Quartiles, Min - Minimum, Max - Maximum
‡P values derived from *Kruskal-Wallis* chi-squared test and were adjusted (Bonferroni) for multiple comparisons. The null hypothesis is that data distribution is homogenous. P values ≤0.05 reject the null hypothesis.

Table S4. Descriptive statistics of gastrointestinal tracts organs lengths (mm) of animals randomly selected from each Experimental group (Control – CON and Treatment -TRT) for postmortem and histopathology investigation. .

Organ	Group CON (n = 3)			Group TRT (n = 3)			P value ‡
	Mean (SD)	Median (Q1, Q3)	Min, Max	Mean (SD)	Median (Q1, Q3)	Min, Max	
**Jejunum**	142 (70)	160 (112, 181)	65, 202	506 (636)	148 (139, 694)	130, 1240	0.51
**Ileum**	20317 (2218)	21070 (19440, 21560)	17820, 22060	91243 (68892)	121300 (66860, 130600)	12430, 140000	0.51
**Caecum**	188 (83)	165 (142, 222)	120, 280	151 (14)	155 (145, 158)	135, 162	0.28
**Colon**	767 (225)	820 (670, 890)	520, 960	530 (269)	680 (450, 685)	220, 690	0.82

Key: SD - Standard Deviation, Q1 & Q3 - First and Third Quartiles, Min - Minimum, Max - Maximum
‡P values derived from *Kruskal-Wallis* chi-squared test and were adjusted (Bonferroni) for multiple comparisons. The null hypothesis is that data distribution is homogenous. P values ≤0.05 reject the null hypothesis.

Table S5. Descriptive statistics of organ tissue fold density[€] and length of animals randomly selected from each Experimental group (Control – CON and Treatment -TRT) for postmortem and histopathology investigation. .

Variable	Group CON (n = 3)			Group TRT (n = 3)			P value‡
	Mean (SD)	Median (Q1, Q3)	Min, Max	Mean (SD)	Median (Q1, Q3)	Min, Max	
**Tissue fold density**	1.5 (0.5)	2.0 (1.0, 2.0)	1.0, 2.0	2.1 (0.8)	2.0 (2.0, 3.0)	1.0, 3.0	0.09
**Tissue fold length**	1.3 (0.5)	1.0 (1.0, 2.0)	1.0, 2.0	1.8 (0.8)	2.0 (1.0, 2.0)	1.0, 3.0	0.03

Key: SD - Standard Deviation, Q1 & Q3 - First and Third Quartiles, Min - Minimum, Max – Maximum

€ Fold density grading scheme used: Grade 1 = minimal findings; Grade 2 Moderate findings; Grade 3 Marked findings.
‡ P values derived from *Kruskal-Wallis* chi-squared test and were adjusted (Bonferroni) for multiple comparisons. The null hypothesis is that data distribution is homogenous between the Groups. P values ≤0.05 reject the null hypothesis.

Table S6. Organ histopathology microscopic villi length (mm) and width (mm) histopathological measurement animals randomly selected from Control (CON) and Treatment (TRT) for postmortem and histopathology investigation. .

Organ	Group CON			Group TRT			P value ‡
	**Mean (SD)**	**Median (Q1,Q3)**	**Min, Max**	**Mean (SD)**	**Median (Q1, Q3)**	**Min, Max**	
**Villi Length (mm)**							
Rumen Atrium	1.46 (0.79)	1.31 (0.78,1.90)	0.05,3.32	1.69 (0.90)	1.54 (1.10, 2.02)	0.11, 4.37	0.28
**Rumen Ventral Sac**	**1.31 (0.39)**	**1.33 (1.04, 1.53)**	**0.45, 2.17**	**1.75 (0.67)**	**1.69 (1.28, 2.27)**	**0.42, 3.24**	**0.04**
Rumen Blind Sac	1.59 (1.02)	1.28 (0.76, 1.98)	0.51, 4.27	1.35(0.77)	1.33 (0.64, 1.89)	0.04, 4.44	0.23
Omasum	4.25 (4.59)	1.85 (1.28, 4.83)	0.81,17.36	6.33(9.52)	1.84 (0.80, 8.62)	0.12,40.73	0.28
Reticulum	3.04 (2.35)	3.34 (0.85, 4.28)	0.43,9.15	2.29 (1.89)	1.60 (0.84, 3.21)	0.44, 7.55	0.32
Duodenum	0.66 (0.22)	0.57 (0.47,0.84)	0.38, 1.13	0.75 (0.28)	0.66 (0.57,0.90)	0.12, 1.43	0.23
**Jejunum - Proximal**	0.86 (0.28)	0.80 (0.72, 0.98)	0.46, 1.79	1.16 (0.50)	1.26 (0.67,1.50)	0.31, 2.18	0.32
Jejunum - Middle	**1.32 (0.35)**	**1.37 (0.98, 1.63)**	**0.65, 1.89**	**0.87 (0.22)**	**0.84 (0.72, 0.94)**	**0.34, 1.61**	**0.03**
Jejunum - Distal	0.71 (0.18)	0.73 (0.64,0.84)	0.33, 1.12	0.83 (0.21)	0.85 (0.73, 0.99)	0.09, 1.44	0.13
**Ileum**	**0.62 (0.12)**	**0.60 (0.55, 0.67)**	**0.33, 1.10**	**0.80 (0.18)**	**0.80 (0.72, 0.91)**	**0.05, 1.13**	**0.00**
Colon	0.47 (0.06)	0.48 (0.45, 0.52)	0.27, 0.56	0.52 (0.11)	0.50 (0.46, 0.60)	0.33, 0.75	0.48
Caecum	0.51 (0.10)	0.52 (0.41, 0.58)	0.29, 0.78	0.53 (0.14)	0.49 (0.43, 0.63)	0.28, 0.82	0.38
Abomasum	0.55 (0.32)	0.44 (0.34, 0.60)	0.20, 1.40	0.59 (0.21)	0.49 (0.43, 0.72)	0.36, 1.08	0.36
**Villi Width (mm)**							
Rumen Atrium	0.28 (0.07)	0.28 (0.22,0.32)	0.17, 0.47	0.37 (0.27)	0.31 (0.13, 0.57)	0.05, 0.98	0.31
**Rumen Ventral Sac**	**0.22 (0.11)**	**0.23 (0.14,0.29)**	**0.02, 0.54**	**0.37 (0.12)**	**0.37 (0.26, 0.46)**	**0.15, 0.62**	**0.06**
Rumen Blind Sac	0.24 (0.14)	0.20 (0.16, 0.30)	0.07, 0.78	0.34 (0.15)	0.31 (0.23, 0.43)	0.07, 0.70	0.23
**Omasum**	**0.66 (0.18)**	**0.68 (0.53,0.79)**	**0.11, 1.02**	**0.42, (0.14)**	**0.40 (0.34, 0.47)**	**0.13, 1.01**	**0.02**
Reticulum	0.43 (0.18)	0.35 (0.29, 0.59)	0.17, 0.85	0.43 (0.29)	0.34 (0.19, 0.57)	0.10, 1.45	0.23
Duodenum	0.05 (0.01)	0.05 (0.04, 0.06)	0.02, 0.08	0.06 (0.0)	0.05 (0.04, 0.08)	0.02, 0.12	0.17
Jejunum - Proximal	0.05 (0.02)	0.05 (0.04, 0.06)	0.03, 0.10	0.08 (0.12)	0.06 (0.05, 0.07)	0.03, 0.82	0.37
Jejunum - Middle	0.04 (0.01)	0.04 (0.03, 0.05)	0.01, 0.08	0.51 (0.65)	0.09 (0.06, 1.19)	0.04, 1.97	0.20

Jejunum - Distal	0.06 (0.03)	0.06 (0.04, 0.08)	0.02, 0.18	0.07 (0.10)	0.06 (0.05, 0.07)	0.03, 0.83	0.38
**Ileum**	0.10 (0.15)	0.07 (0.05, 0.10)	0.03, 0.90	0.08 (0.05)	0.06 (0.05, 0.10)	0.03, 0.25	0.45
**Colon**	**0.07 (0.07)**	**0.06 (0.04, 0.07)**	**0.02, 0.55**	**0.09 (0.10)**	**0.08 (0.06, 0.09)**	**0.03, 0.83**	**0.02**
**Caecum**	**0.05 (0.02)**	**0.05 (0.04, 0.07)**	**0.03, 0.09**	**0.07 (0.02)**	**0.07 (0.05,0.08)**	**0.03, 0.11**	**0.07**

‡ P values derived from *Kruskal-Wallis* chi-squared test and were adjusted (Bonferroni) for multiple comparisons. The null hypothesis is that data distribution is homogenous between the Groups. P values ≤0.05 reject the null hypothesis.

Table S7. Organ histopathology microscopic villi surface area (μm) of animals randomly selected from each Experimental group (Control – CON and Treatment -TRT) for postmortem and histopathology investigation. .

**Organs**	**Group CON**			**Group TRT**			P values ‡
	**Mean (SD)**	**Median (Q1,Q3)**	**Min ,Max**	**Mean(SD)**	**Median (Q1,Q3)**	**Min ,Max**	
**Villi surface area (μm)**							
Rumen atrium	0.81 (0.71)	0.52 (0.35, 1.04)	0.17, 3.53	0.95 (1.07)	0.59 (0.37, 1.02)	0.10, 6.57	0.23
Rumen ventral Sac	0.54 (0.49)	0.39 (0.22, 0.66)	0.11, 2.63	0.94 (0.82)	0.70 (0.45, 1.24)	0.15, 4.19	0.12
Rumen blind Sac	1.21 (0.94)	1.01 (0.38, 1.79)	0.11, 3.27	0.67 (0.64)	0.44 (0.32, 0.74)	0.10, 3.32	0.18
Omasum	8.13 (7.25)	4.62 (3.02, 11.49)	0.49, 35.64	6.85 (6.12)	5.15 (2.05, 8.90)	0.04, 24.02	0.26
Reticulum	1.90 (2.39)	0.83 (0.33, 2.15)	0.11, 9.12	1.93 (1.99)	1.32 (0.64, 2.95)	0.11, 13.64	0.26
**Duodenum**	**0.38 (0.13)**	**0.39 (0.29, 0.45)**	**0.12, 0.68**	**0.50 (0.21)**	**0.47 (0.36, 0.69)**	**0.10, 0.87**	**0.08**
Jejunum - proximal	0.53 (0.21)	0.50 (0.36, 0.74)	0.10, 0.98	0.48 (0.22)	0.43 (0.33, 0.60)	0.05, 0.98	0.33
**Jejunum - middle**	**0.50 (0.21)**	**0.50 (0.32, 0.62)**	**0.14, 0.94**	**0.42 (0.17)**	**0.40 (0.33, 0.50)**	**0.13, 0.97**	**0.09**
Jejunum - distal	0.40 (0.22)	0.43 (0.19,0.58)	0.10,0.95	0.55 (0.24)	0.55 (0.37, 0.71)	0.10, 1.09	0.29
Ileum	0.49 (0.20)	0.54 (0.40, 0.60)	0.11, 0.88	0.48 (0.20)	0.44 (0.35, 0.63)	0.11, 0.93	0.28
Colon	0.29 (0.14)	0.27 (0.22, 0.32)	0.13, 0.96	0.32 (0.15)	0.29 (0.21, 0.39)	0.12, 0.95	0.35
Caecum	0.34 (0.16)	0.32 (0.24, 0.40)	0.11, 0.95	0.37 (0.15)	0.35 (0.25, 0.46)	0.17, 0.78	0.15
Abomasum	0.32 (0.11)	0.28 (0.25, 0.36)	0.18, 0.67	0.37 (0.17)	0.31 (0.23, 0.49)	0.12, 0.84	0.25

‡ P values derived from *Kruskal-Wallis* chi-squared test and were adjusted (Bonferroni) for multiple comparisons. The null hypothesis is that data distribution is homogenous between the Groups. P values ≤0.05 reject the null hypothesis.

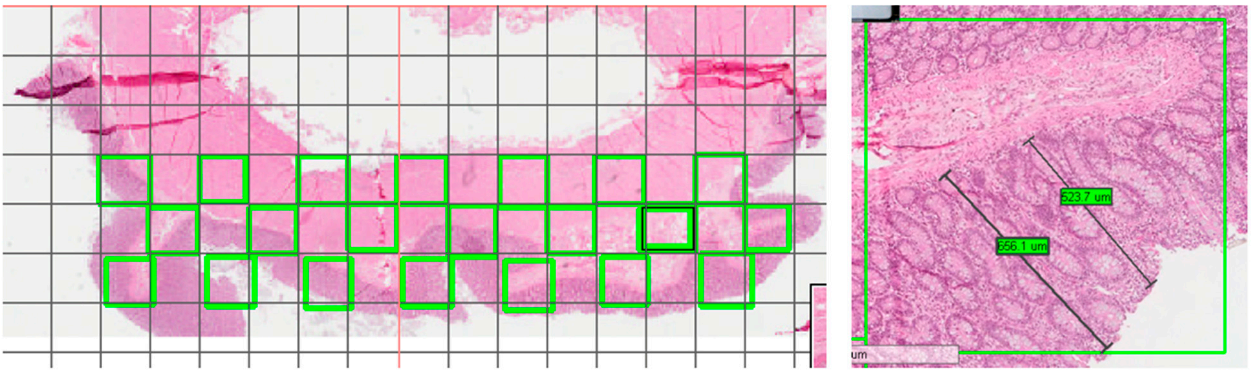

Figure S1. An example of area selection for linear measurement forestomach (reticulum, rumen, omasum). The linear measurements were obtained through measurement of all intact villi as shown in Figure S2

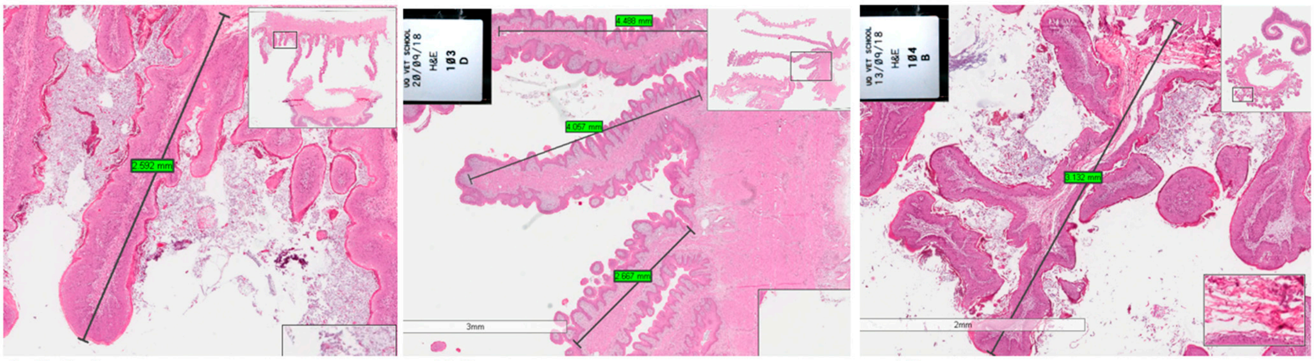

a) Reticulum b) Omasum c) Rumen

Figure S2. Linear measurements of forestomach example.
